# The Barriers and Motivators to Using Human Tissues for Research: The Views of UK-Based Biomedical Researchers

**DOI:** 10.1089/bio.2019.0138

**Published:** 2020-08-05

**Authors:** Emma Lawrence, Jessica Sims, Amir Gander, Jonathan M. Garibaldi, Barry Fuller, Brian Davidson, Philip R. Quinlan

**Affiliations:** ^1^ Surgery and Interventional Science, University College London Medical School, London, United Kingdom.; ^2^ School of Computer Science, University of Nottingham, Nottingham, United Kingdom.; ^3^ Digital Research Service, University of Nottingham, Nottingham, United Kingdom.

**Keywords:** researcher attitudes, discovery of samples, peer network resources, quality

## Abstract

***Introduction:*** The use of human-derived samples is vital to numerous areas of biological and medical research. Despite this, researchers often find or anticipate difficulty in sourcing samples. There are ongoing efforts to increase the visibility and accessibility of UK human tissue biobanking, but minimal (if any) research on the reasons behind researchers' choice of sample source has been undertaken. We have analyzed UK researchers' motivations on using their preferred sample sources and their perceived barriers to human sample use.

***Methods:*** The study was based on an online survey of academic and industry researchers, followed by focus groups, with participants across the United Kingdom. Both the survey and focus groups probed participants' views on the barriers to finding and using human samples in research.

***Results:*** One hundred ninety-eight academic and industry researchers completed the survey on their human sample use, and five focus groups consisting of 21 total participants took place. The top cited reasons for choosing sources included the availability of linked clinical data (40%), the geographical location of the resource (39%), and preexisting collaboration (33%). Focus group participants highlighted their strong preference for local or known sample sources, which were preferred because additional scientific and logistical input could be obtained for their work and they were more confident that the samples would be of good quality.

***Discussion:*** We found that there were significant perceptions of governance barriers to sample access. As a consequence, researchers preferred local and known suppliers because of the perception that these could assist with the governance, would be reliable, and able to provide the additional support they required. Equally, data availability was a major contributor to the selection of a new source of samples. These observations are of significant value to those seeking to improve the access to existing sample resources via online discovery tools.

## Introduction

Human samples are used across the biomedical research spectrum in a multitude of disciplines and sectors. It has been predicted that the demand for human samples will increase with the development of precision or stratified medicine. As such, a 2013 report by the Academy of Medical Sciences stated that there should be an increase in the collection of tissues for biomarker research and that the samples should be stored in a biobank.^1^ Therefore, the need for human samples in biomedical research will remain, and will continue to increase, in the coming years.

The concern, however, is that despite the recognition of their importance, there remains difficulties in accessing human samples for research,^2–4^ even though there are many ways in which the samples can be acquired or sourced. Many of these issues were highlighted in a 2011 report published by a consortium of UK medical researcher funders.^5^ In response to these difficulties, both European^6^ and national initiatives^7^ have been established. The aim of these efforts is to facilitate the location of samples and reduce duplication in collections. Online directories, portals, and discovery platforms have been created to try and allow researchers to find samples and sample sources, or biobanks, more easily. These platforms can assist both in the discovery of existing samples and resources that could collect new samples for a specific need.

Previous work on making samples and sample sources findable has been based on data standards that have focused on the samples,^8,9^ the quality of the sample^10^ or different consent models and permissions.^11^ The directories have then provided a search layer on top of these standards. The focus of these directories is presenting an accurate description of the samples held in the biobanks. These efforts have clearly aided the ability of biobanks and the samples to be found. However, the creation of directories has not solved the issue of several research communities who still report challenges in finding suitable human tissue samples for their research. Thus, it is important that directories take into consideration the behavior, needs, and wants of biomedical researchers to ensure utilization of the samples that have been donated for research, collected and archived in UK Biobanks. Despite the importance of research using human samples, there is little documented understanding of how biomedical researchers source samples and the factors that influence their choices.

The authors are also responsible for the development of the UK's Tissue Directory, and as such wanted to ensure that we understood the factors behind sample source choice so that we could adapt and enhance our own work and also provide invaluable context to a world-wide challenge. We embarked on research to better understand the human sample landscape in the UK, with a particular focus on the attitudes of researchers toward the use of online directories. This was not an evaluation of any single directory, but an exercise to understand how researchers currently access samples and how an online system could assist in this process. However, we want to utilize the results of this work to understand the implications for those developing directories or methods for researchers to find and access new sources of samples.

## Methods

The study was based on a survey, followed by focus group sessions. Ethical approval for this research was granted by the UCL Research Ethics committee (REC) on January 17, 2018. The Project ID and Title number is 12303/001: Barriers to using an online directory to identify human samples for biomedical research. The approval covered both the running of an online survey and the in-person focus groups. Inclusion criteria were that all UK-based Post-PhD Biomedical researchers are invited to partake in this study. “Biomedical” referred to the study of any area of science with a basic or translational medical application. Exclusion criteria were anyone without a PhD, based outside of the UK or who studied plant or environmental Biology. This work does not separate different types of users (e.g., academic vs. industry). The goal was to gather a broad representation on the views of researchers currently utilizing samples in their research.

The survey, “Human samples in UK Biomedical research,” was conducted between January and February 2018, while the focus group meetings were held between August and November 2018. The methods for advertising both aspects of the studies included posting to social media, advertising in internal and external newsletters, and circulating the invitation among existing project collaborators. Focus group invitations were also extended to willing volunteers who had completed the online survey.

### Survey

The questions were entered into the SurveyMonkey platform using set logic. The following were four sections of questions related to different aspects of the respondents, their experimental models, and their motivations: (1) About you (questions about the respondents), (2) Experimental models (questions about the models used in their research), (3) Sourcing human samples, and (4) Barriers to sample access.

### Focus groups

The focus groups began with the facilitator giving a brief outline of what the research was seeking to achieve. The participants were also informed that (1) the recordings would be transcribed, but that the transcripts would be kept in anonymous form, (2) the transcripts would only be seen by members of the research team and would then be analyzed for key themes that had arisen from the discussion, and (3) the data will be used in publications and participants can opt into updates on these. Finally, a Research Ethics Committee-approved focus group topic guide was prepared and followed.

## Results

### Survey results

**FIG. 1.**
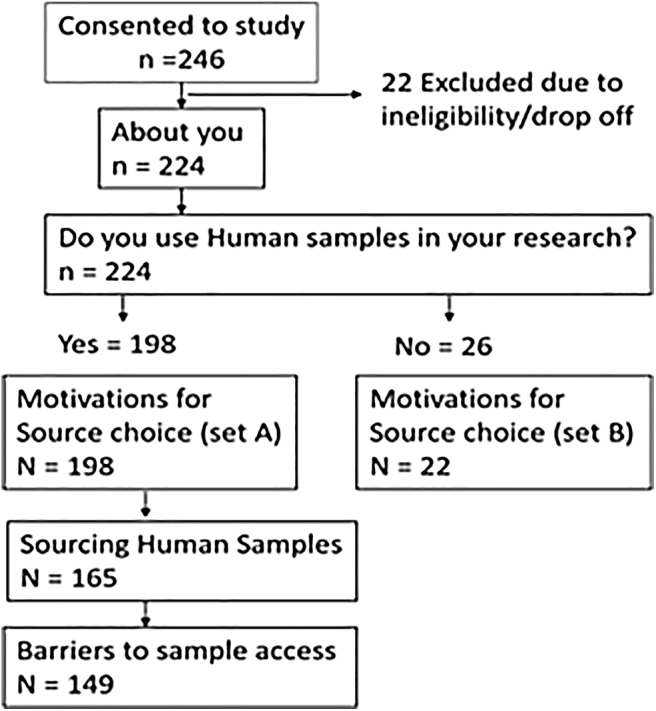
Detailing the study recruitment numbers and how they changed over the different stages of the survey.

Two hundred forty-six researchers consented to take part, 22 were excluded from analysis due to ineligibility, and 26 were excluded as the researchers were not using human samples in their research (Fig. 1). The analysis therefore represents 198 survey responses with the majority of respondents being a Principal/Senior Investigator or at the group head level. The respondents who cited “Other” came from a variety of positions, including managers, research nurses, and other roles, in industry. Respondents could select more than one field of research interest. The most frequently cited research interest (74 respondents) was oncology and the least cited field was “Evolutions, systems and genomics” (5 respondents).

### Current access

**FIG. 2.**
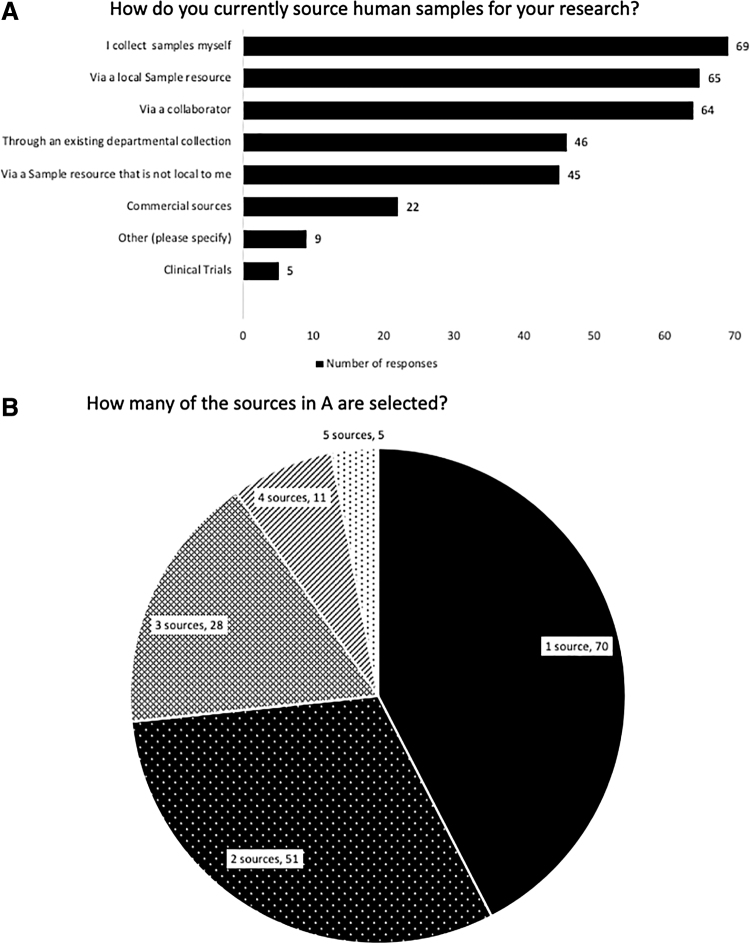
**(A)** The percentage of respondents who sourced samples via a particular method. **(B)** The breakdown on the percentage of respondents who used a varying number of sample sources for their research.

**FIG. 3.**
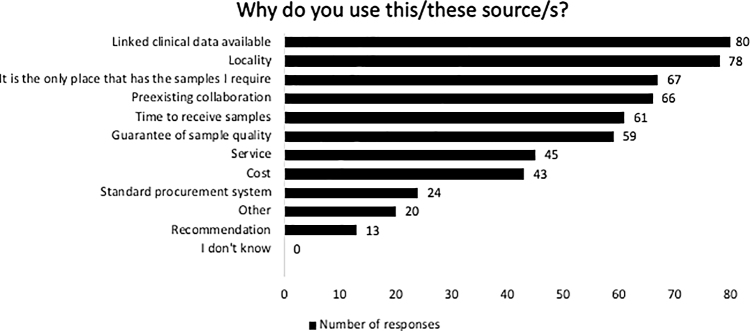
The reasons why the respondents selected their current source of samples.

Of the 198 respondents who used human samples, a variety of different sample sources were identified (Fig. 2A). Participants could select more than one answer, and indeed over half of participants used more than one source for their samples (Fig. 2B). The most popular methods of sample acquisition were self-collection, via a local sample resource and via a collaborator. The top cited reasons for their chosen source were the availability of linked clinical data (*n* = 80, 48%) (Fig. 3), followed by the location of the resource (*n* = 78, 47%), and it is the only place that has the samples I require (*n* = 67, 41%).

### Barriers to sample access

**FIG. 4.**
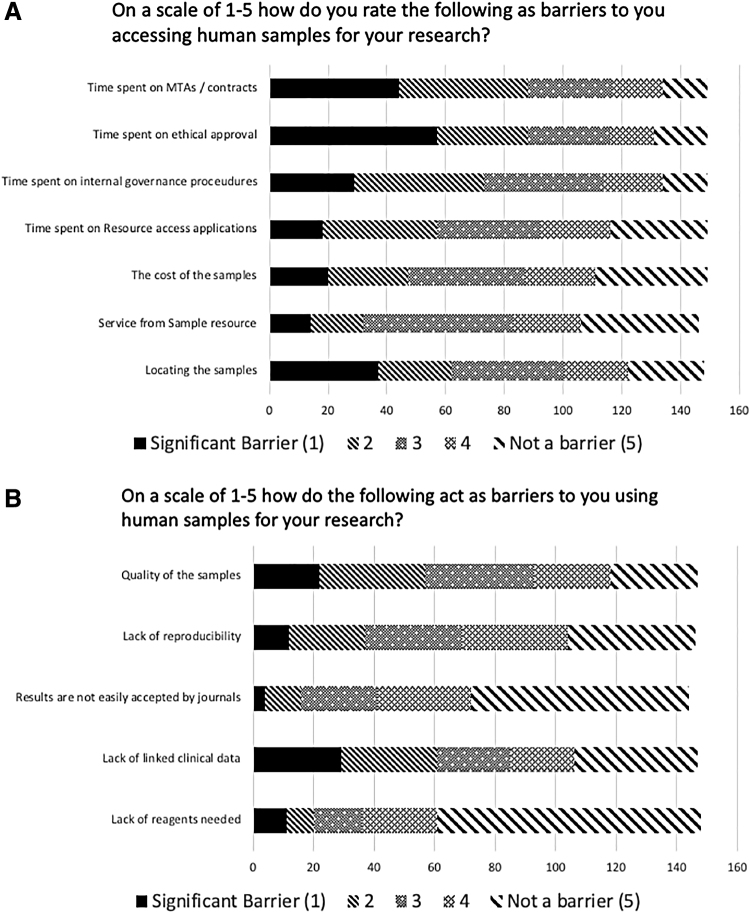
The breakdown and scale of barriers to accessing samples **(A)** and using samples **(B)** in research.

**FIG. 5.**
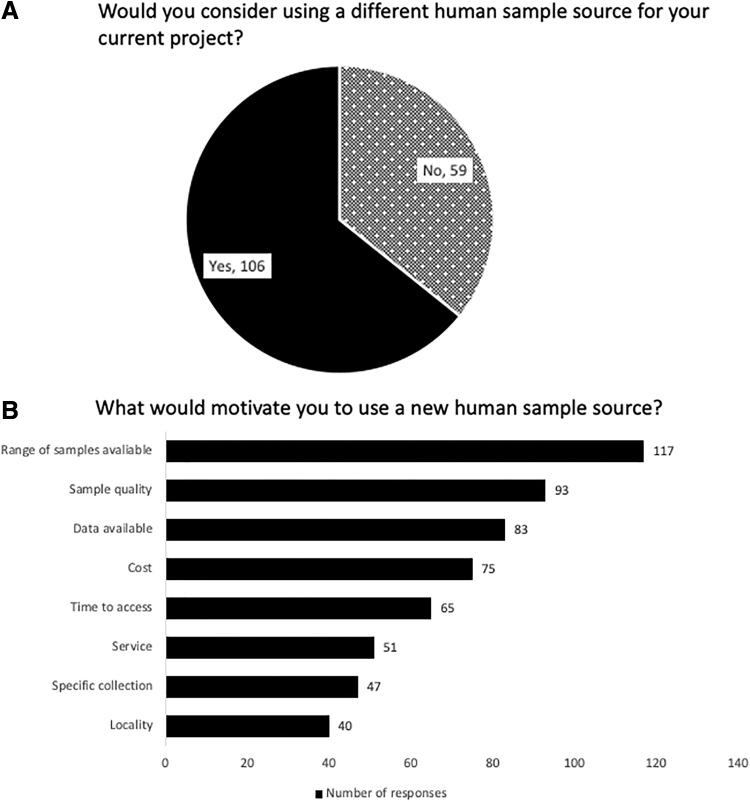
The percentage of respondents who would consider using a new source **(A)** and the motivators for using a new source of samples **(B)**.

Respondents could rate different barriers, with 1 (the left side of the Figures) being a “significant barrier” through to “not a barrier” (the right of the Figures), the stages in between represent gradients of that position. “Time spent on MTAs [Material Transfer Agreements] and contracts” and “Time spent on ethical approval” were cited as the biggest barriers to sample access (Fig. 4A), with nearly 60% (88/149 for both questions) of respondents citing them to be 1 (significant) to 2 (high) barriers (Fig. 4B). The largest barrier to the use of samples in research was the “Lack of linked clinical data” with 42% (61/147) of respondents ranking it as either a 1 (significant) or 2 (high) barrier. Nearly two-thirds (106/165) of respondents said that they would consider using a different human sample source for their current project (Fig. 5A). There was no significant difference between the answers to Figure 2A and the willingness to use a different source for future projects. The most popular factors for selecting a new sample source were the range of samples and the quality of the samples (Fig. 5B).

### Focus groups

Five focus groups featuring a total of 12 researchers were held around the country (Table 1) and the discussions were analyzed resulting in the themes discussed below.

**Table 1.** Detailing the Dates and Attendees at the Focus Group Events

Focus group	Date	No. of participants	Duration	Transcript size (words)
1	August 1, 2018	6	1:56:07	17,950
2	October 18, 2018	4	1:32:18^a^	11,342
3	October 3, 2018	4	1:55:55	15,297
4	November 17, 2018	5	1:44:10	11,519
5	November 6, 2018	2	1:33:38	13,697
Total		12		

^a^ Partial recording loss due to technical difficulties.

#### Personal relationships

The theme of building and maintaining trusted relationships arose as important in the sample sourcing process throughout the focus groups. One specific theme within this was that personal contact with the sample provider would be desired. For example, participants felt that it would be easier to work with people with whom they had built up a rapport:

“I think it's easier if you have a named person because it feels more personal…. and I think once you have a contact and built a rapport with someone it's much easier…” (P5D).

In these instances, the actual source did not matter, regardless of whether the source was a collaborator, biobank, or local clinical team, as long as rapport was established with a named contact and preferably through face-to-face interaction. As a consequence, many participants expressed a preference for local sources so that these relationships could be initiated, developed, and maintained more easily:

“… you can talk to somebody face-to-face, you can pick up the samples—you've not got to worry about transportation—and it's the ease of communication” (P4B).

In general, nonlocal resources would only be considered if personal contact with a named person could be established early in the process.

#### Governance and access

The focus group participants felt that, even for an established researcher, processes and procedures vary from institution to institution, which adds to the complexity of working with human samples. It became evident in focus group discussions that participants used local relationships as a proxy for guidance on formal and informal governance processes in their institution, and as a way of mitigating the risk if anything goes wrong with sample acquisition.

Participants voiced concerns that even after investing time and resources into sample access applications, the samples may not actually be released for use because of contractual or ethical restrictions that are only discovered later in the process. Participants indicated that they would trust the reliability of the provider if they had used them as a source before:

“…yeah, there's definitely a difference I would say between the two different types: … you've got the, ‘what you know’, ‘what you're safe with’, ‘what you've done before’, or, ways that you can see things growing…” (P2E).

Participants often referenced either returning to a previous source or tapping into an existing network that could provide referrals to a trusted source. The use of either their own or a colleague's relationship was, therefore, used as a way of assessing the reliability of the resource.

“…someone will be like: whatever you do, don't go with them; they were a nightmare to deal with… I think you're going to go with your colleague's experiences on these.” (P6A).

#### Collaboration

In addition to information about processes and procedures, participants often referenced the need for scientific input from their sample source. This was also perceived as a way of increasing the likelihood of successful research. Receiving associated scientific input was the most common theme cited of the usefulness of working with a sample provider. These included the need for guidance on a particular sample type or technique, the need for guidance in experimental design, and the desire to have help with analysis, article preparation, and underwriting research with additional credibility.

“…going from completely preclinical, I don't have any contact with patients or clinicians and now I see that it's possible for me to do my experiments on human tissue. But, there's a bunch of support and expertise that I need around that to help deliver good quality science…” (P1C).

The desire for research support arose not just from wanting successful experiments for scientific outputs, but also to avoid the wastage of samples that would arise from unsuccessful experiments:

“…otherwise it's just meaningless and it's a waste of tissue which is not really acceptable so it's good to have a proper chat to make sure you've thought about everything…” (P2C).

#### Quality

Participants also felt that building relationships was key to maximizing the chance of getting samples, which were of the right standard for the research. Relationships would therefore result in a sense of trust in the sample provider delivering on the request.

The participants cited that they would rely on their sample provider to give accurate assessments of sample quality and some found it difficult to quantify quality outside of trust and relationships. A source's reputation, a colleague's experience, or direct previous interaction was therefore considered as a way to ascertain a source's ability to deliver samples of good quality. In total, eight participants from four focus groups made reference to trust and sample quality. Citations in publications were another way of developing trust in the sample provider's credentials and track record.

“They may have published in the area so you've some degree of trust that they're going to be…have the quality and interest in the field that you're doing.” (P1E).

There was a noticeable lack of references to formal assessments of sample quality, such as biobank accreditation or use of nationally or internationally recognized standards throughout all focus group discussions.

## Discussion

The use of human tissue samples in biomedical research remains a complex and heterogeneous sector involving many aspects, including patient altruistic donation and ethics, the need for a complex system for tissue collection, the service aspects of biobank collection and storage, and the underlying driver of providing appropriate and high-quality human tissue to academic and commercial researchers. Although the tissue provision aspects have received considerable focus, the implications of the current tissue provision system for individual researchers have received minimal research effort. This project is the first of its kind to seek to understand the current mechanism in which sample sources are found to understand how efforts such as the UKCRC Tissue Directory and Coordination Centre can make an impact on the well-reported challenge of accessing human samples for research.

The survey, which looked at current practice, found that researchers still accessed samples by predominately local mechanisms (either a local biobank, collect themselves, or via a collaborator). The focus groups confirmed that this was not simply by chance, but a strong preference of researchers, as it was seen as a way of building rapport and trust to facilitate a reliable and high-quality contribution to a research program. The self-collection was the single most popular option, which does demonstrate that this approach is still prevalent despite the proliferation of sample resources and the reported underutilization. It is clear from these results that researchers are seeking to find a trusted research collaborator to help guide them through the complex area on topics such as governance, ethics, study design, sample quality, and suitability for the research proposed. This perception of complexity must be considered as a key driver for those who opt for self-collection, as this group was no more or less likely to be open to utilizing a different source of samples in the future, but must revert to self-collection when the alternatives are perceived to be too complex and time consuming. Directories alone cannot solve this issue and a coordinated effort must be undertaken to either streamline access processes and procedures and also to ensure governance myths, such as those that surround the recent European Union General Data Protection Regulation are addressed.

The (poor) quality of samples came out as one of the major barriers to utilizing samples and (good) quality samples a motivator for accessing a new source of samples. However, the focus group results demonstrate a wide spectrum of concepts that researchers relate to quality, that are not necessarily the formal concepts that people within the biobanking community would consider. Indeed, quality was also one of the aspects that researchers would seek support from the sample resource to advise on the suitability of samples for different uses. The results would suggest that the concept of quality can change at different points of the discovery and sample use journey. When initially seeking new sample resources, quality appeared to be more about the overall research standing of the resource, such as if any notable researcher had cited their use or a particular resource. Therefore, it was clear that the peer network is of significant importance when assessing a potential new source of samples, as a way to gathering soft intelligence on the likely quality of the resource. Researchers did acknowledge that one of the core reasons for approaching a sample resource is for very specific advice and support on whether their planned use of samples would be appropriate, with an indirect acknowledgement that they do not necessarily understand what samples can be used for in all circumstances. This would be evidenced by a general lack of reference to quality measures such as from the International Standards Organization or European Committee for Standardization. Therefore, we must be careful not to assume that quality has a universal meaning, and in fact different criteria for quality will exist at different stages of the discovery pathway, from finding a suitable sample resource to the use of samples for a specific purpose.

The availability of clinical data was also a significant driver in why the researchers were using their current source, and the lack of clinical data being cited as the largest barrier for samples to be used in research. This is maybe not a surprise given success stories such as UK Biobank, which have focused so strongly on data and suggestions that biobanks should adopt a “data first” approach to ensure that the datasets are available before collecting samples. The ability for sample resources to link to and access clinical data is potentially the biggest challenge still to face this community, as the requirement for ever richer datasets places a new digital requirement onto resources that have historically been focused on sample collection activities.

### Implications for directories

Online directories that facilitate the discovery of sample resources have generally been created by the community, with a focus on correctly representing a sample resource in a data form. The Minimum Information About BIobank data Sharing (MIABIS) data standard^8^ can represent the contents of a sample resource and many (if not all) of the Directories developed in Europe are based on MIABIS. This work has focused on what researchers value when seeking to find a new sample resource. While data standards such as MIABIS can give an accurate representation of the samples and collections a sample resource may hold, it does not capture the “research excellence” components that researchers desire, such as the citations and wider use of the sample resource in the community, or the desire to find a collaborator rather than a collection. This is not a criticism of MIABIS or other similar work (much of which has been undertaken by the authors), but the results of the survey and focus groups suggest that making more detailed data about samples visible will not necessarily result in researchers finding what they need for their research. Our work suggests that there is a large proportion of researchers seeking colleagues and collaborators to assist them through the challenges of using samples in research. Therefore, it is vital that directories, and data standard efforts such as MIABIS, feature the research community's’ current methods and desires for finding a new sample resource and are not simply inventories of samples. This work suggests that they should equally be defined by the expertise and skills of the staff that run and coordinate them alongside the samples and data they can provide.

## Conclusion

To those engaged in research, it may not be surprising to reveal the importance placed on collaboration, reputation, and trust. Access to samples remains a challenge for a large portion of the research community, and efforts to address this access challenge via directories must understand and adopt these aspects. Researchers are neither seeking a transactional arrangement for samples nor searching on sample properties, but seeking the research experience and expertise of those working at the sample resources to support and develop their research.

## References

[1] The Academy of Medical Sciences oversight group. (2013). Realising the potential of stratified medicine. The Academy of Medical Sciences. https://acmedsci.ac.uk/viewFile/51e915f9f09fb.pdf

[2] Edwards J, Belvisi M, Dahlen S-E, Holgate S, Holmes A Human tissue models for a human disease: What are the barriers? Thorax 2015;70:695 25631322 10.1136/thoraxjnl-2014-206648 PMC4483787

[3] Massett HA, Atkinson NL, Weber D, et al. Assessing the need for a standardized cancer human biobank (caHUB): Findings from a national survey with cancer researchers. JNCI Monogr 2011;2011:8–15 10.1093/jncimonographs/lgr007 21672890

[4] use My data, Incisive Health and Medicines Discovery Catapult. (2020). The Issue with Tissue: Recommendations for improving the use of human tissue samples. Medicines Discovery Catapult. http://www.usemydata.org/resources/11027_THE%20ISSUE%20WITH%20TISSUE_REPORT_A4_DIGITAL.pdf

[5] UK Clinical Research Collaboration (UKCRC) Experimental Medicine Funders Group and The NCRI's Board Sub-group on Clinical Translational Research. (2011). UK Funders' Vision for Human Tissue Resources. UK Clinical Research Collaboration. https://www.ukcrc.org/wp-content/uploads/2014/03/Vision+for+human+tissue+resources.pdf

[6] Mayrhofer MT, Holub P, Wutte A, Litton J-E BBMRI-ERIC: The novel gateway to biobanks. From humans to humans. Bundesgesundheitsblatt Gesundheitsforschung Gesundheitsschutz 2016;59:379–384 26860601 10.1007/s00103-015-2301-8

[7] Quinlan PR, Lawrence E, Pourabdolla A, et al. The UK Clinical Research Collaboration (UKCRC) tissue directory and coordination centre: The UK's centre for facilitating the usage of human samples for medical research. Open Journal of Bioresources 2017;4:6

[8] Loreana N, Fransson MN, Eriksson M, et al. A minimum data set for sharing biobank samples, information, and data: MIABIS. Biopreserv Biobank 2012;10:343–348 24849882 10.1089/bio.2012.0003

[9] Quinlan PR, Mistry G, Bullbeck H, Carter A A data standard for sourcing fit-for-purpose biological samples in an integrated virtual network of biobanks. Biopreserv Biobank 2014;12:184–191 24785371 10.1089/bio.2013.0089 PMC4066222

[10] Lehmann S, Guadagni F, Moore H, et al. Standard preanalytical coding for biospecimens: Review and implementation of the sample preanalytical code (SPREC). Biopreserv Biobank 2012;10:366–374 24849886 10.1089/bio.2012.0012 PMC6463986

[11] Woolley JP, Kirby E, Leslie J, et al. Responsible sharing of biomedical data and biospecimens via the “Automatable Discovery and Access Matrix” (ADA-M). NPJ Genom Med 2018;3:17 30062047 10.1038/s41525-018-0057-4 PMC6056554

